# Maternal dietary ratio of linoleic acid to alpha-linolenic acid during pregnancy has sex-specific effects on placental and fetal weights in the rat

**DOI:** 10.1186/s12986-018-0330-7

**Published:** 2019-01-03

**Authors:** Sally A. V. Draycott, Ge Liu, Zoe C. Daniel, Matthew J. Elmes, Beverly S. Muhlhausler, Simon C. Langley-Evans

**Affiliations:** 1 Food and Nutrition Research Group, Department of Food and Wine Science, School of Agriculture Food and Wine, University of Adelaide, Adelaide, Australia; 2 School of Biosciences, University of Nottingham, Sutton Bonington Campus, Loughborough, UK; 3 Healthy Mothers, Babies and Children’s Theme, South Australian Health and Medical Research Institute, North Terrace, Adelaide, 5001 Australia

**Keywords:** Maternal nutrition, Fatty acids, Animal model, Fetal growth

## Abstract

**Background:**

Increased consumption of linoleic acid (LA, omega-6) in Western diets coupled with the pro-inflammatory and adipogenic properties of its derivatives has led to suggestions that fetal exposure to this dietary pattern could be contributing to the intergenerational cycle of obesity.

**Method:**

This study aimed to evaluate the effects of maternal consumption of a LA to alpha-linolenic acid (ALA) ratio similar to modern Western diets (9:1) compared to a lower ratio (1:1.5) on placental and fetal growth, and to determine any cumulative effects by feeding both diets at two total fat levels (18% vs 36% fat w/w). Female Wistar rats (*n* = 5–7/group) were assigned to one of the four experimental diets prior to mating until 20d of gestation.

**Results:**

Fatty acid profiles of maternal and fetal blood and placental tissue at 20d gestation were different between dietary groups, and largely reflected dietary fatty acid composition. Female fetuses were heavier (2.98 ± 0.06 g vs 3.36 ± 0.07 g, *P* < 0.01) and male placental weight was increased (0.51 ± 0.02 g vs 0.58 ± 0.02 g, *P* < 0.05) in the low LA:ALA groups. Female fetuses of dams exposed to a 36% fat diet had a reduced relative liver weight irrespective of LA:ALA ratio (7.61 ± 0.22% vs 6.93 ± 0.19%, *P* < 0.05). These effects occurred in the absence of any effect of the dietary treatments on maternal bodyweight, fat deposition or expression of key lipogenic genes in maternal and fetal liver or maternal adipose tissue.

**Conclusion:**

These findings suggest that both the total fat content as well as the LA:ALA ratio of the maternal diet have sex-specific implications for the growth of the developing fetus.

## Introduction

The prevalence of obesity, a major risk factor for cardiovascular disease and type 2 diabetes, continues to rise in both low-, middle- and high-income countries, with 1.9 billion adults worldwide estimated to be overweight or obese as of 2016 [1]. Of particular concern is that 41 million children under the age of 5 were estimated to be overweight or obese globally in 2016 [1], and often present with early onset of cardiometabolic diseases including type 2 diabetes and hypertension [2, 3].

Exposure to either an inappropriately high or inappropriately low plane of nutrition before birth and/or in early infancy is a major risk factor for the development of obesity and type 2 diabetes through the life course [4–7]. Maternal high-fat diets have consistently been associated with an increased risk of obesity and poor cardiometabolic health in the offspring [8]. It is increasingly recognised, however, that the impact of maternal fat consumption is dependent not only on the amount of fat consumed but also the specific fat type. The ratio of omega-6 and omega-3 polyunsaturated fatty acids (PUFA) in the diet appears to be of particular importance, likely due to the opposing roles of these two classes of PUFA on metabolic and inflammatory processes within the body [9]. Thus, omega-3 PUFA, including alpha-linolenic acid (ALA) and its long chain PUFA (LCPUFA) derivatives, eicosapentaenoic acid (EPA) and docosahexaenoic acid (DHA) inhibit inflammation, adipogenesis and lipogenesis [10, 11]. Conversely, the omega-6 PUFA, linoleic acid (LA) and its long chain derivative, arachidonic acid (AA) have pro-inflammatory actions and promote adipogenesis and lipogenesis in vitro and in vivo [12, 13]. Consequently, excess consumption of omega-6 relative to omega-3 fatty acids would be expected to be associated with increased incidence of inflammatory conditions, increased adiposity and heightened risk of cardiometabolic diseases.

The opposing roles of the omega-3 and omega-6 fatty acids has particular relevance due to a substantial increase in the intake of the omega-6 essential fatty acid, LA, in the Western diet over the past few decades, with very little change in the intake of omega-3 PUFA. The ratio of LA:ALA in typical Western diets has been reported at ~ 8–10:1 [14, 15] or higher [16], which is substantially higher than the proposed ‘ideal’ ratio of ~ 1–2:1 based on levels required to achieve maximal conversion of ALA to its longer chain derivatives [17–19]. Previous studies have shown that the high omega-6:omega-3 ratio in the modern Western diets is reflected in the blood and tissue fatty acid profiles of pregnant and lactating women, as well as breast milk [13], however, the effect of perinatal exposure to an increased omega-6:omega-3 PUFA ratio remains unclear. Massiera [20] demonstrated that increasing omega-6 PUFA intake induced a gradual enhancement in fat mass over generations. However, the level of LA (~ 19% energy) and LA:ALA ratio (28:1) in their study were much higher than those found in typical Western diets, and the impact of different dietary fat levels was not evaluated. In addition, no studies to date have examined the effects of an increased maternal omega-6:omega-3 ratio on maternal, placental and fetal outcomes before birth.

The aim of the current study was to determine the effects of increasing the LA:ALA ratio (9:1 vs 1:1.5), in the diet of rats, on maternal weight gain, placental and fetal growth and the expression of lipogenic and adipokine genes in maternal and fetal liver and maternal adipose tissue. To determine any additive effects of altering the maternal dietary LA:ALA ratio each diet was fed at either 18% fat w/w, to reflect dietary recommendations of fat intake [21], or at a higher fat content of 36% fat w/w.

## Materials and methods

### Animals

All animal procedures were performed in accordance with the Animals (Scientific Procedures) Act 1986 under Home Office licence and were approved by the Animal Ethics Committee of the University of Nottingham, UK. Virgin female Wistar rats (*n* = 24; 75-100 g; Charles River, UK) were housed on wood shavings in individually ventilated cages under a 12 h light/12 h dark cycle at a temperature of 20–22 °C and had ad libitum access to food and water throughout the experiment. Animals were pair housed from the start of the experiment until mating, after confirmation of conception animals were individually housed until completion of the experiment. Female rats were allowed to acclimatise to the unit for 1–2 weeks, during which time they were fed on standard laboratory chow (2018 Teklad Global 18% Protein Rodent Diet, Harlan Laboratories, UK). After acclimatisation, a tail vein blood sample was taken from each animal for the determination of fatty acid status. The rats were then randomly assigned to one of 4 dietary groups, details of which are provided below. Animals were maintained on their allocated diet for a four week ‘feed-in’ period after which they were mated. Conception was confirmed by the presence of a semen plug and this was recorded as day 0 of pregnancy. Average time in mating pairs was 2.5 days and all but one female conceived within their first oestrus cycle housed with the male. Female rats remained on their respective diets until day 20 of gestation (full term = 22 days) at which time rat dams were euthanised by CO_2_ asphyxiation and cervical dislocation and fetuses by cervical dislocation and exsanguination for collection of maternal, fetal and placental tissues. All female rats were weighed and had feed intake measured daily throughout the experiment and protein intake was estimated based on the protein content of the experimental diets.

### Diets

Diets were designed to provide either a high (9:1, high LA) or low (1:1.5 low LA) ratio of LA to ALA, achieved by altering the amounts of flaxseed and sunflower oil included in the fat component of the feed. The levels of saturated fatty acids (SFA) and monounsaturated fatty acids (MUFA) were comparable in all diets, achieved by adjusting the amounts of coconut (SFA source) and macadamia (MUFA source) oils in the diets. For each level of LA, diets containing either 18% or 36% fat by weight were developed. This resulted in four experimental diets; high LA (18% fat), high LA (36% fat), low LA (18% fat) and low LA (36% fat) (*n* = 5–7 per dietary group). The list of ingredients and final fatty acid composition of the four experimental diets is presented in Table 1 and Table 2 respectively. Protein content of experimental feeds was analysed using a Thermo Scientific nitrogen (N)/protein analyser (Flash EA1112). %N was directly measured and protein (%) was estimated under the assumption of a 16% N content of proteins.

**Table 1:** Ingredients in the Experimental Diets

Component	Amount (g/100 g diet)			
	High LA (18% Fat)	High LA (36% Fat)	Low LA (18% Fat)	Low LA (36% Fat)
Casein	16.0	16.0	16.0	16.0
Cornflour	31.8	20.0	31.8	20.0
Sucrose	16.0	10.0	16.0	10.0
Cellulose	15.6	15.6	15.6	15.6
Mineral Mix^a^	1.8	1.8	1.8	1.8
Vitamin Mix^a^	0.4	0.4	0.4	0.4
Choline Chloride	0.2	0.2	0.2	0.2
Methionine	0.4	0.4	0.4	0.4
Flaxseed Oil	0.9	1.7	4.2	8.5
Sunflower Oil	6.6	13.1	0.8	1.7
Macadamia Oil	6.9	13.8	9.5	19.0
Coconut Oil	3.4	6.9	3.2	6.3
Total Fat	17.8	35.6	17.8	35.5

*LA* linoleic acid. ^a^Composition of vitamin and mineral mixes based on AIN-76 formulation [50]

**Table 2:** Fatty Acid Composition of the Experimental Diets

Fatty Acid	Amount (% of total lipids)			
	High LA (18% Fat)	High LA (36% Fat)	Low LA (18% Fat)	Low LA (36% Fat)
Total SFA	20.7	20.3	21.1	20.0
Total TFA	0.1	0.1	0.1	0.1
Total MUFA	48.8	49.0	53.3	54.9
Total Omega-6 (LA only)	27.3	27.4	11.1	10.5
Total Omega-3 (ALA only)	3.1	3.2	14.4	14.4
Total PUFA	30.4	30.6	25.5	25.0
LA:ALA Ratio	9.0	9.0	0.75	0.75
ALA %Energy	1.2	2.0	5.6	9.1
LA %Energy	10.5	17.3	4.3	6.7

*LA* linoleic acid, *SFA* saturated fatty acids, *TFA* trans fatty acids, *MUFA* monounsaturated fatty acids, *ALA* alpha-linolenic acid, *PUFA*, polyunsaturated fatty acids

### Blood sample and tissue collection

Blood samples were collected from dams prior to the start of the experiment and after the 4 week ‘feed-in’ period (tail vein) and at day 20 of gestation (cardiac puncture following CO_2_ asphyxiation and cervical dislocation). Truncal blood samples were also collected from two randomly selected male and two randomly selected female fetuses from each litter at this time. The whole blood samples (~ 30 μl) were spotted onto PUFAcoat™ dried blood spot (DBS) collection paper [22], allowed to dry at room temperature and were then stored at − 20 °C for subsequent fatty acid analysis. Maternal tissues were weighed and samples of liver and retroperitoneal adipose tissue were collected. All fetuses were weighed and sexed via measurement of anogenital distance. Fetal liver and placentas were weighed and samples of selected male placental and liver samples were collected. All tissue samples were snap-frozen in liquid nitrogen and stored at − 80 °C until determination of gene expression by reverse transcriptase quantitative PCR (RT-qPCR). Male fetuses selected for collection of tissue samples also had tail samples collected for sex-genotyping by PCR for the SRY gene [23]. Any samples found to be female or inconclusive (*n* = 5) were not included in hepatic gene expression analyses.

### Lipid extraction

Total lipids were extracted from male placental tissue with chloroform/isopropanol (2:1 v/v), an adapted method from Folch [24]. Briefly, ~ 300 mg of crushed frozen placenta was weighed out and 2 ml of 0.9% saline was added. The solution was homogenised on ice, 3 ml of isopropanol was added and vortexed and the solution was left to stand for 5 min. After this, 6 ml of chloroform was added and the solution was shaken, centrifuged and the chloroform (bottom phase) containing the lipid was transferred into a fresh tube. This was dried under nitrogen and resuspended in chloroform/methanol (9:1 v/v) and 20 μl of the sample was then spotted onto PUFAcoat™ DBS collection paper [22], allowed to dry at room temperature and stored at − 20 °C for subsequent fatty acid analysis.

### Fatty acid methylation and analysis

Fatty acid composition in maternal and fetal DBS were determined as previously described [22]. Briefly, whole DBS samples were directly transesterified with 2 ml of 1%H_2_SO_4_ in methanol at 70 °C for 3 h. Samples were allowed to cool and the fatty acid methyl esters (FAME) were then extracted with heptane and transferred into a glass vial containing anhydrous sodium sulphate (Na_2_SO_4_) as a desiccant. Samples were separated and analysed by a Hewlett-Packard 6890 gas chromatograph (GC) equipped with a capillary column (30 m × 0.25 mm) coated with 70% cyanopropyl polysilphenylene-siloxane (BPX-70) (0.25 μm film thickness) which was fitted with a flame ionization detector (FID). Helium was utilised as the carrier gas and the split ratio was set at 20:1. Injector temperature was set at 250 °C and FID temperature at 300 °C. FAMEs were identified in unknown samples based on the comparison of retention times with an external lipid standard (Standard 463, Nu-check prep Inc., MN, USA) using Agilent Chemstation software. Individual fatty acid content was calculated based on peak area and response factors normalised to total fatty acid content and expressed as a percentage of total fatty acids across lipid classes.

### Isolation of RNA, cDNA synthesis and reverse transcription quantitative real-time PCR (RT-qPCR)

RNA was isolated from crushed snap-frozen samples of ~ 25 mg of liver using the Roche High Pure Tissue kit (Roche Diagnostics Ltd., UK). Adipose RNA was extracted using MagNA lyser green beads and instrument (Roche Diagnostics Ltd.) in combination with the RNeasy Mini Kit (QIAGEN Ltd., UK) with the following modifications. Between 100 and 150 mg of snap frozen tissue was homogenised using green beads in 600 μl Buffer RTL (including β-mercaptoethanol), the sample was centrifuged for 3 min at 8000 rpm and the resulting infranatant was transferred into a fresh tube. The remaining tissue sample was homogenised in a further 350 μl of lysis buffer, centrifuged as before and this infranatant was combined with that from the initial extraction. This extract was centrifuged for 5 min at 13000 rpm, cleared lysate removed and mixed with an equal volume of 70% ethanol and transferred to an RNeasy column and from this point the standard manufacturer’s protocol was applied. RNA concentration was determined using a Nanodrop 2000 (Thermo Scientific) and RNA quality was evaluated by agarose gel electrophoresis. cDNA was synthesised using a RevertAid™ reverse transcriptase kit (Thermo Fisher Scientific, UK) with random hexamer primers.

Lipogenic pathway and adipokine target genes included; peroxisome proliferator-activated receptor gamma (*Pparg*), sterol regulatory element-binding protein (variant 1c) (*Srebf1*), fatty acid synthase (*Fasn*), lipoprotein lipase (*Lpl*) and leptin (*Lep)*, with β-actin (*Actb*) as the housekeeper (Table 3). Adipocyte and hepatic gene expression was quantified using SYBR Green (Roche Diagnostics) in a Light-Cycler 480 (Roche Diagnostics). Samples were analysed against a standard curve of a serially diluted cDNA pool to produce quantitative data and expression was normalised to the housekeeping gene using LightCycler® 480 software (version 1.5.1) as previously described [25]. The expression of the housekeeper was not different between treatment groups.

**Table 3:** Primer sequences used for the determination of gene expression by RT-qPCR

Target Genes	Sequence (5′-3′)	Amplicon Size	Accession Number
*Fasn*	FWD: TGCTCCCAGCTGCAGGC	107	NM_017332
	REV: GCCCGGTAGCTCTGGGTGTA		
*Lpl*	FWD: TTCCTGGATTAGCAGACTCTGTGT	89	NM_012598
	REV: TCCTGTCACCGTCCATCCAT		
*Pparg*	FWD: CTCAGTGGAGACCGCCCA	75	NM_013124
	REV: CAGGGCCTGCAGCAGGT		
*Srebf1*	FWD: GATTGCACATTTGAAGACATGCTT	95	NM_001276708
	REV: CCTGTCTCACCCCCAGCATA		
*Lep*	FWD: AGACCATTGTCACCAGGATCAAT	89	NM_013076
	REV: CCCGGGAATGAAGTCCAAA		
*Actb*	FWD: GACCCAGATCATGTTTGAGACCTT	79	NM_031144
	REV: AGAGGCATACAGGGACAACACA		

*Fasn* fatty acid synthase, *Lpl* lipoprotein lipase, *Pparg* peroxisome proliferator-activated receptor gamma, *Srebf1* sterol regulatory element-binding protein 1, *Lep* leptin, *Actb* beta-actin

### Statistical analysis

Data are presented as mean ± SEM. Data were analysed using the Statistical Package for Social Sciences (Version 24, SPSS Inc.). The effect of maternal dietary fatty acid ratio and maternal dietary fat content on maternal dependant variables was assessed using a two-way ANOVA, with dietary fat level and LA:ALA ratio as factors. Where longitudinal data was analysed, as with maternal feed, protein and energy intake as well as maternal bodyweight, the impact of maternal dietary fat level and LA:ALA ratio was analysed using a two-way repeated-measures ANOVA. Fetal data were analysed using a two-way ANOVA, separating males and females, with maternal dietary LA:ALA ratio and fat content as factors. The experiment was designed to detect a 0.5 g difference in fetal weight with a power of 0.8, a value of *P* < 0.05 was considered to be statistically significant and the dam was used as the unit of analysis.

## Results

### Maternal food intake

**Fig. 1:**
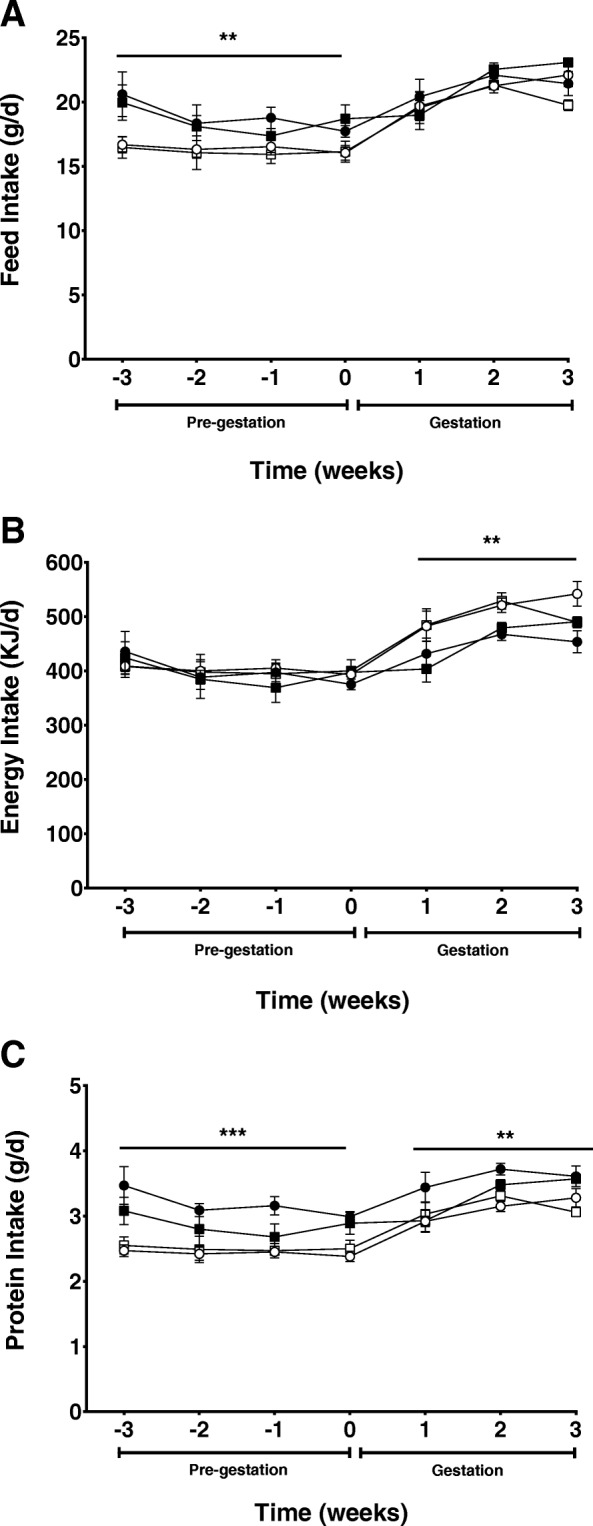
Maternal average daily (**a**) food intake, (**b**) energy intake and (**c**) protein intake before mating and up to day 20 of gestation fed on either a high LA (18% fat) diet (closed circles), high LA (36% fat) diet (open circles), low LA (18% fat) diet (closed squares) and a low LA (36% fat) diet (open squares). Values are means ± SEM and *n* = 5–7 per group. The effects of dietary fatty acid ratio and dietary fat content were determined using a two-way repeated measures ANOVA. * indicates a significant effect of dietary fat content (** *P* < 0.01, *** *P* < 0.001)

Dams receiving the 36% fat diets had a significantly lower food intake in the 4 weeks prior to mating independent of the LA:ALA ratio (*P* < 0.01). During pregnancy there was no effect of dietary fat content or fatty acid ratio on maternal food intake although, as expected, food intake in all groups increased as pregnancy progressed (*P* < 0.05; Fig. 1a). Energy intake was similar between groups prior to pregnancy. During gestation energy intake was higher in dams consuming the 36% fat diets, irrespective of dietary LA:ALA ratio (*P* < 0.01). Similar to food intake, energy intake also increased with increasing gestational age in all dietary groups (*P* < 0.05; Fig. 1b). Protein intake was lower in the 36% fat groups when compared to the 18% fat groups prior to mating (*P* < 0.001) and throughout gestation (*P* < 0.01). Protein intake was relatively consistent for the 4 weeks prior to mating but increased with increasing gestational age during pregnancy (*P* < 0.05) (Fig. 1c). There was no significant effect of dietary LA:ALA ratio on food, energy or protein intake at any time during the study.

### Maternal fatty acid profile

**Fig. 2:**
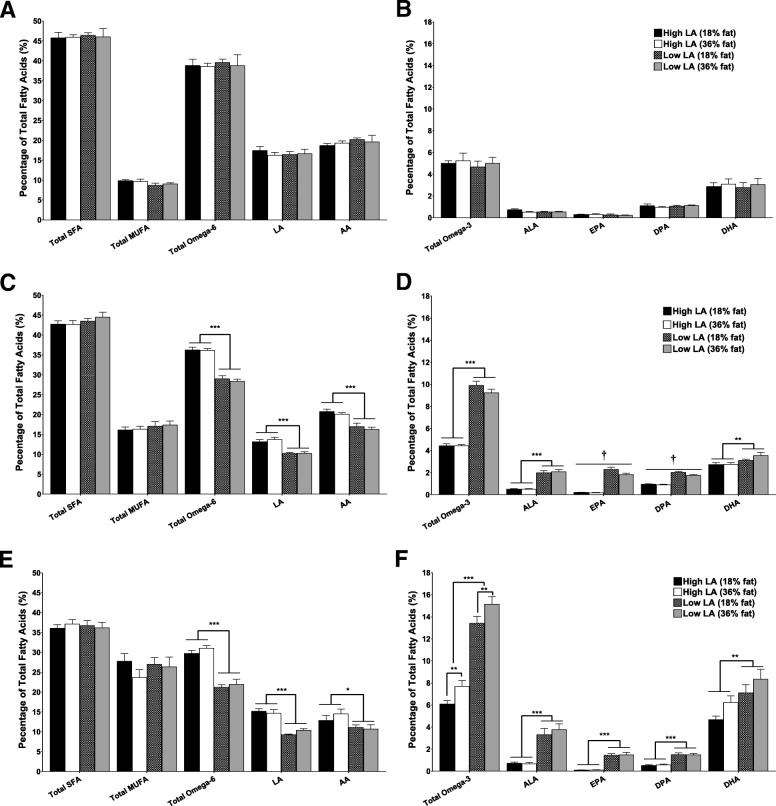
Maternal whole blood fatty acids profile at (**a**/**b**) baseline (**c**/**d**) after 4 weeks on experimental diet and (**e**/**f**) at day 20 of gestation. Values are means ± SEM and *n* = 5–7 per group. The effects of dietary fatty acid ratio and dietary fat content were determined using a two-way ANOVA (*P < 0.05, **P < 0.01, ***P < 0.001). † indicates a significant interaction between dietary fat content and LA:ALA ratio (*P* < 0.05)

There were no differences in the blood fatty acid profiles of the rats assigned to the different experimental diets before commencement of the dietary intervention, and the proportions of SFA, MUFA, omega-6 (Fig. 2a) and omega-3 PUFA (Fig. 2b) were all similar between groups. After 4 weeks on their respective diets, however, maternal fatty acid profiles were significantly different between treatments and largely reflected the composition of the experimental diets. Whole blood proportions of LA and AA were lower in the low LA compared to the high LA groups (*P* < 0.001; Fig. 2c). Dams fed the low LA diets had four-fold higher ALA levels compared to the high LA groups, independent of dietary fat content (*P* < 0.001). Whole blood proportions of DHA were also significantly higher (*P* < 0.01) in dams fed the low LA diet, but the magnitude of this effect was relatively small (1.3-fold; Fig. 2d). The proportion of EPA was ten-fold higher and DPA two-fold higher in dams consuming the low LA diets. However, relative EPA and DPA levels were both lower in the low LA, 36% fat diet group compared to the low LA, 18% fat group, but were not different between the 36 and 18% fat diet levels in rats fed the high LA diets (EPA: fat content, *P* < 0.05; fatty acid ratio, *P* < 0.001; interaction, *P* < 0.05. DPA: fat content, *P* < 0.01; fatty acid ratio, *P* < 0.001; interaction, *P* < 0.05) (Fig. 2d). There was no effect of dietary fat content on the proportions of EPA and DPA at the end of the experiment. The remaining effects of both total fat level and dietary fat ratio on maternal fatty acid profile were, however, maintained until the end of the study (day 20 of gestation) (Fig. 2e and f).

### Maternal weight and body composition

**Fig. 3:**
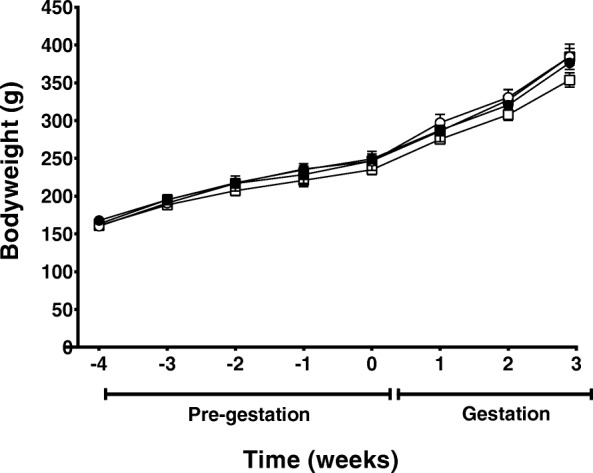
Maternal weight gain of rats during pre-feeding and up to day 20 of gestation fed on either a high LA (18% fat) diet (closed circles), high LA (36% fat) diet (open circles), low LA (18% fat) diet (closed squares) and a low LA (36% fat) diet (open squares). Values are means ± SEM and *n* = 5–7 per group. The effects of dietary fatty acid ratio and dietary fat content were determined using a two-way repeated measures ANOVA and no significant differences were found between groups

Bodyweight immediately prior to commencement of feeding the experimental diets was not different between rats assigned to the 4 respective treatment groups (Fig. 3). There was no effect of either LA:ALA ratio or dietary fat content on maternal weight prior to mating or throughout pregnancy (Fig. 3) and maternal weight at day 20 of pregnancy was similar between groups (Fig. 3; Table 4). Dams in the low LA groups had larger hearts relative to body weight at day 20 of gestation (*P* < 0.05), but there were no differences in the relative weight of other major organs, including the liver, kidney, lung and brain, between groups (Table 4). There were no significant differences in the mass of the gonadal and retroperitoneal fat depots between experimental groups (Table 4). There were also no differences in the expression of any of the genes measured in the hepatic or adipose tissue between dietary groups (Table 4).

**Table 4:** Maternal organ weights and gene expression

	High LA (18% Fat)	High LA (36% Fat)	Low LA (18% Fat)	Low LA (36% Fat)
Bodyweight (g)	376.4 ± 4.65	384.41 ± 11.00	384.34 ± 16.90	353.63 ± 9.55
Heart (% BW)	0.25 ± 0.01^a^	0.26 ± 0.01^a^	0.28 ± 0.02^b^	0.28 ± 0.01^b^
Lungs (% BW)	0.37 ± 0.05	0.36 ± 0.03	0.38 ± 0.05	0.36 ± 0.04
Kidney (%BW)	0.53 ± 0.02	0.50 ± 0.01	0.53 ± 0.02	0.54 ± 0.02
Liver (% BW)	4.01 ± 0.14	3.95 ± 0.07	4.10 ± 0.10	3.94 ± 0.10
Brain (% BW)	0.47 ± 0.02	0.46 ± 0.01	0.50 ± 0.03	0.53 ± 0.02
Gonadal Fat (% BW)	2.95 ± 0.03	2.38 ± 0.18	2.33 ± 0.18	3.04 ± 0.58
Retroperitoneal Fat (% BW)	1.18 ± 0.17	1.18 ± 0.17	1.18 ± 0.011	1.79 ± 0.24
Liver mRNA Expression				
*Fasn*	5.51 ± 1.33	2.83 ± 0.59	6.08 ± 2.31	4.24 ± 1.21
*Lpl*	3.13 ± 1.00	2.39 ± 0.75	2.52 ± 0.80	1.74 ± 0.71
*Pparg*	5.81 ± 1.85	2.30 ± 0.77	3.38 ± 0.78	2.09 ± 0.73
*Srebf1*	1.33 ± 0.31	1.20 ± 0.22	1.23 ± 0.50	2.67 ± 1.14
Retroperitoneal Fat mRNA Expression				
*Fasn*	0.12 ± 0.05	0.07 ± 0.04	0.15 ± 0.05	0.13 ± 0.07
*Lpl*	2.18 ± 0.66	1.82 ± 0.81	2.18 ± 0.72	1.48 ± 0.16
*Pparg*	1.15 ± 0.47	1.40 ± 0.59	1.76 ± 0.48	1.03 ± 0.17
*Srebf1*	1.17 ± 0.40	1.16 ± 0.41	1.38 ± 0.42	1.11 ± 0.13
*Lep*	2.59 ± 0.67	2.73 ± 0.99	2.31 ± 0.69	1.83 ± 0.34

Data are presented as mean values ± SEM (*n* = 4–6 per group). *LA* linoleic acid, *BW* bodyweight, *mRNA* messenger ribonucleic acid, *Fasn* fatty acid synthase, *Lpl* lipoprotein lipase, *Pparg* peroxisome proliferator-activated receptor gamma, *Srebf1* sterol regulatory element-binding protein 1, *Lep* leptin. Mean values with unlike superscript letters were significantly different (*P* < 0.05)

### Placental fatty acid profile

**Fig. 4:**
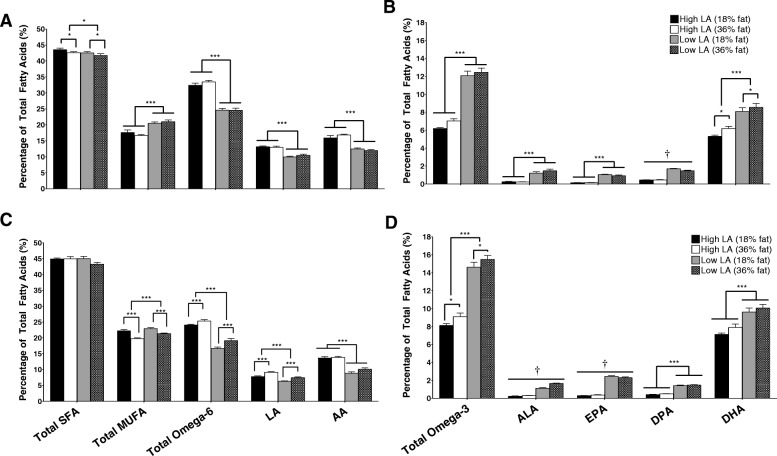
Placental (**a**/**b**) and fetal whole blood (**c**/**d**) fatty acid profile at gestational day 20. Values are means ± SEM and *n* = 10–14 per group. The effects of maternal dietary fatty acid ratio and maternal dietary fat content were determined using a two-way ANOVA (*P < 0.05, **P < 0.01, ***P < 0.001). † indicates a significant interaction between dietary fat content and LA:ALA ratio (*P* < 0.05)

The placental fatty acid profile at day 20 of gestation (Fig. 4a and b) was similar to dam whole blood fatty acid profile at this time point (Fig. 2e and f). Placentas of dams fed a high LA diet had 1.3 fold higher LA, AA and total omega-6 PUFA irrespective of dietary fat content (*P* < 0.001; Fig. 4a). Conversely, total omega-3 PUFA (1.9 fold), ALA (5.5 fold) and EPA (6.5 fold) proportions were increased in placentas of dams consuming a low LA diet, regardless of dietary fat content (*P* < 0.001). The relative levels of DPA in the placenta were higher in dams receiving a low LA diet but were also influenced by total dietary fat content, such that the proportion of DPA was lower in dams fed the 36% vs. 18% fat diets in the low LA ratio groups only (fat content, *P* < 0.01; fatty acid ratio, *P* < 0.001; interaction, *P* < 0.01). DHA proportions were also influenced by both the fat content and LA:ALA ratio of the diet, such that dams exposed to the low LA diets had higher placental DHA compared to those fed on high LA diets (*P* < 0.001) and dams fed the 36% fat diet had higher relative levels of placental DHA compared to dams consuming either of the 18% fat diets (*P* < 0.05; Fig. 4b). Placental total MUFA proportions were higher in the low LA compared to the high LA groups (*P* < 0.001) but were not affected by dietary fat content. Placental SFA were lower in dams exposed to a low LA diet (*P* < 0.05) as well as in dams exposed to a high (36%) fat diet (*P* < 0.05), however the magnitude of this difference was very small (Fig. 4a).

### Fetal whole blood fatty acid profile

There was no effect of sex on fetal fatty acid profile and so male and female samples were combined for subsequent analysis. Blood ALA (five-fold) and EPA (seven-fold) were higher in fetuses of dams exposed to the low LA diets. However, there was a significant interaction between maternal dietary LA:ALA ratio and maternal dietary fat content for these fatty acids, such that increasing dietary fat content from 18 to 36% fat was associated with relatively higher ALA (fat content, *P* < 0.001; fatty acid ratio, *P* < 0.001; interaction, *P* < 0.001) and lower EPA levels (fat content, *P* = 0.721; fatty acid ratio, *P* < 0.001; interaction, *P* < 0.05) in the low LA groups only. DPA (three-fold) and DHA (1.3-fold) blood proportions were also increased in fetuses of dams exposed to the low LA diets, independent of dietary fat content (*P* < 0.001; Fig. 4d). Conversely, fetuses of dams consuming the low LA diets had relatively lower circulating LA (1.2-fold) and AA (1.4-fold) compared to fetuses of dams fed high LA diets (*P* < 0.001; Fig. 4c). Interestingly, fetal LA proportions were also influenced by dietary fat content, such that fetuses of dams consuming the 36% fat diets had higher relative circulating LA (*P* < 0.001) levels compared to fetuses of dams fed the 18% fat diet. Fetal blood MUFA were influenced by both maternal dietary LA:ALA ratio and dietary fat content. Thus, fetuses of dams exposed to a low LA diet exhibited elevated MUFA proportions compared to high LA groups (*P* < 0.001) and fetuses of dams exposed to a 36% fat diet had proportionately lower circulating MUFA levels compared to fetuses of dams fed the 18% fat diets (*P* < 0.001; Fig. 4c).

### Fetal and placental weight and hepatic gene expression

The impact of maternal dietary fat content and LA:ALA ratio on placental and fetal weights was different for males and females. Thus, placental weight was higher in male, but not female, fetuses of dams consuming low LA diets (*P* < 0.05). In females, however, fetuses of dams consuming the low LA diet were heavier (*P* < 0.01) than those of dams fed the high LA diets, irrespective of dietary fat content, this effect was not seen in males. Despite these differences in fetal and placental weights, however, the fetal-placental weight ratio was not different between treatment groups in either males or females. Female, but not male, fetuses of dams consuming 36% fat diets also had smaller livers relative to bodyweight, independent of the dietary LA:ALA ratio (*P* < 0.05; Table5). Hepatic gene expression was not assessed in female fetuses, but hepatic mRNA expression of *Fasn, Lpl, Pparg* and *Srebf1* in male fetuses was not different between groups (Table 6).

**Table 5:** Fetal and placental weights and fetal organs

	Sex	High LA (18% Fat)	High LA (36% Fat)	Low LA (18% Fat)	Low LA (36% Fat)
Fetal bodyweight (g)	Male	3.33 ± 0.04	3.25 ± 0.08	3.55 ± 0.09	3.32 ± 0.09
	Female	3.01 ± 0.06^a^	2.95 ± 0.09^a^	3.36 ± 0.12^b^	3.21 ± 0.08^b^
Placenta weight (g)	Male	0.50 ± 0.02^a^	0.52 ± 0.03^a^	0.56 ± 0.03^b^	0.61 ± 0.02^b^
	Female	0.48 ± 0.02	0.48 ± 0.04	0.52 ± 0.02	0.51 ± 0.03
Fetal liver weight (% BW)	Male	7.43 ± 0.42	6.52 ± 0.11	6.81 ± 0.58	6.97 ± 0.25
	Female	7.51 ± 0.38^a^	6.99 ± 0.29^b^	7.73 ± 0.22^a^	6.86 ± 0.28^b^
Fetal-placental ratio	Male	6.79 ± 0.24	6.37 ± 0.33	6.51 ± 0.41	5.99 ± 0.41
	Female	6.38 ± 0.32	6.35 ± 0.48	7.72 ± 0.96	6.13 ± 0.36

Data are presented as mean values ± SEM (*n* = 4–6 per group). *LA* linoleic acid, *BW* bodyweight. Mean values with unlike superscript letters were significantly different (*P* < 0.05)

**Table 6:** Fetal Liver Gene expression

	High LA (18% Fat)	High LA (36% Fat)	Low LA (18% Fat)	Low LA (36% Fat)
*Fasn*	0.59 ± 0.19	0.30 ± 0.03	0.37 ± 0.06	0.49 ± 0.13
*Lpl*	1.79 ± 0.55	1.41 ± 0.27	1.11 ± 0.33	1.17 ± 0.46
*Pparg*	0.36 ± 0.12	0.42 ± 0.05	0.34 ± 0.07	0.48 ± 0.16
*Srebf1*	1.21 ± 0.61	0.65 ± 0.30	0.27 ± 0.04	0.82 ± 0.30

Data are presented as mean values ± SEM (*n* = 4–6 per group). *LA* linoleic acid, *Fasn* fatty acid synthase, *Lpl* lipoprotein lipase, *Pparg* peroxisome proliferator-activated receptor gamma, *Srebf1* sterol regulatory element-binding protein 1

## Discussion

The results of the current study demonstrate that varying the fat content and LA:ALA ratio in the diet during pregnancy results in significant shifts in the fatty acid profile of the dam, placenta and fetus in late pregnancy with dietary LA:ALA ratio eliciting potentially sex-specific effects on placental and fetal weights. Importantly, these effects occurred in the absence of any impact of dietary LA:ALA ratio on maternal energy intake, bodyweight or fat deposition. Doubling the fat content of these experimental diets had the expected impact on maternal food and energy intake but was not associated with any significant alterations in maternal, placental or fetal weights or maternal fat deposition.

Maternal fatty acid profiles after 4 weeks on the experimental diets, and at the end of pregnancy, confirmed that the experimental diets had the desired effect on maternal fatty acid composition. Consistent with previous studies, increasing dietary ALA content had the greatest effect on maternal ALA and EPA proportions whereas relative DHA levels were only slightly higher than controls [26–29]. However, despite the significant shifts in maternal fatty acid profiles, and differences in energy intake between dams in the 36 and 18% fat groups during gestation, we found no difference in maternal body weight or fat deposition between these groups. This was unexpected, previous studies with comparable fat contents have reported significant increases in maternal bodyweight and fat mass in dams fed on diets containing higher amounts of total fat [30, 31]. Consistent with previous studies [32], dams fed on the 36% fat diets in this study reduced their feed intake to compensate for the higher energy density of the feed and it may be that this adaptive response was sufficient to prevent excessive weight gain and fat deposition in dams fed the higher fat diet.

Our finding that placental and fetal fatty acid profiles were similar to those in the dams was consistent with our hypothesis that variations in maternal fatty acid profile would be reflected in the fetal compartment. Thus, our results confirm that shifts in maternal dietary fatty acid intake are associated with corresponding shifts in fetal fatty acid supply. It is, however, important to note that the relative proportions of some fatty acids, most notably LA and MUFA, in the fetal compartment also appeared to be influenced by total dietary fat content, independent of the dietary fat ratio. This effect was not observed to the same extent for maternal and placental fatty acid profiles and implies that not only the ratio, but also absolute amounts of fatty acids is a key determinant of placental fatty acid transfer. Our finding that fetal, but not maternal or placental, LA proportions were higher in the 36% fat vs 18% fat groups implies that placental LA transfer is favoured as dietary fat content increases. Placental capacity to store fatty acids as triglycerides has been well documented [33], and placental triglyceride levels are increased in conditions where maternal circulating triglyceride and fatty acid concentrations are high [34]. However, this storage capacity is limited and in cases of excess maternal dietary fat has the potential to result in a greater proportion of fatty acids being transferred from maternal to fetal circulation. One possibility, therefore, is that the capacity of the placenta for LA storage is exceeded in the 36% fat groups in our current study and that this resulted in a greater amount of this fatty acid being transferred to the fetal compartment. In contrast to LA, however, fetal MUFA proportions in the current study were lower in the 36% fat compared to the 18% fat groups. Thus, the results of our study indicate that the effect of increased dietary fat content on placental fatty acid transfer varies substantially between individual fatty acids.

That the human placenta preferentially transfer specific fatty acids over others, following the hierarchy of AA>DHA > ALA>LA > oleic acid, has been previously demonstrated [35]. The findings from the present study suggest that this selective transfer effect may be exacerbated in the presence of an increased dietary fat load, such that the higher total amounts of LA and ALA in the 36% fat diets lead to greater transfer of LA and ALA to the fetal compartment, at the expense of MUFA, particularly oleic acid. If this is the case, it would imply that the quality of dietary fat intake during pregnancy may be more important, and thus have a greater potential to influence fetal growth and development, at higher levels of total dietary fat intakes. The fatty acid data in our study are expressed as a proportion of total lipids rather than absolute concentrations, and the relatively small proportionate change in fetal LA could translate into a much greater, and physiologically relevant, change in absolute concentrations.

In contrast to previous studies, we found no effect of maternal total fat intake on placental or fetal bodyweight, and our results in fact suggest that maternal LA:ALA ratio had a greater effect on these measures. The increased placental weight of male fetuses in the low LA groups suggests that an increase in maternal omega-3 status may potentially increase placental growth. One possibility for this increase in weight is that omega-3 fatty acids are preferentially deposited in the placenta. This, however, seems unlikely given that previous studies have suggested that while there is preferential placental uptake of DHA from the maternal circulation, the majority is transferred across the placenta to the fetal circulation [35, 36]. In addition, a considerable amount of omega-3 fatty acids (~ 75 mg) would have to accumulate in the placenta to solely account for the difference in placental weight between high and low LA groups, making it unlikely that this mechanism could fully account for the observed increase in placental weight. An alternative possibility is that omega-3 PUFA, in particular DHA, acted to promote placental growth. Why the increased placental weight in our study was only seen in male placentas is not completely clear, however, female placental weight appeared to follow the same trajectory but did not reach statistical significance. Differences in gene expression between male and female placentas have been documented [37].

Given that the effects on placental weight only reached significance in males, it is interesting that maternal consumption of the low LA diet was associated with increased fetal weight only in females. Maternal omega-3 LCPUFA supplementation during pregnancy has been associated with increases in birth weight in human randomised controlled trials [39]. However, this is generally considered to be secondary to an increase in the duration of gestation and therefore cannot explain the higher fetal weights observed in this study, since all fetuses were weighed at the same gestational age. There are, however, previous reports indicating that upregulation of omega-3 LCPUFA-responsive genes following maternal omega-3 LCPUFA supplementation is more pronounced in female compared to male placentas in humans, and is associated with increases in infant birth weight, but not placental weight [38]. Our finding of lower female fetal liver weight in the 36% fat groups is consistent with some [40], but not all [41, 42], studies that have previously reported the effects of maternal high fat diets on neonatal liver weight. Whether the reduced liver weight in fetuses of dams consuming the high fat diet persists after birth, and whether it has functional consequences for hepatic metabolism, remain to be determined.

It was interesting that despite the significant changes in the fatty acid profiles of both dams and fetuses, no changes in maternal (hepatic and adipose tissue) or fetal (hepatic) expression of lipogenic genes were observed in this study. This was despite evidence from previous studies that omega-3 PUFA can supress, while omega-6 PUFA can promote, expression of *Srebf1* and downstream lipogenic genes [43, 44]. It is possible, however, that any changes in maternal gene expression and corresponding increases in lipogenesis could be masked by the normal physiological adaptations that occur during pregnancy, particularly between day 19 and 20 of gestation where the pregnant rat experiences a marked increase in total body fat mass [45]. Similarly, due to the immaturity of hepatic signalling pathways within the fetus, changes in gene expression occurring as a result of altered maternal dietary LA:ALA ratio may be too subtle to be detected.

In conclusion, we have demonstrated that both the total amount of fat and the ratio of LA and ALA in the maternal diet have significant effects on placental and fetal weight, supporting the hypothesis that the quality, as well as the quantity, of fat in the maternal diet can impact on fetal growth and development. Importantly, the LA:ALA ratio in the current study was designed to reflect that of typical Western diets of many countries, and our results show that consuming this high LA diet was associated with alterations in placental and fetal weight in comparison to a LA:ALA ratio considered to be ‘optimal’ for human nutrition [14]. The observed changes in placental and fetal weight may contribute to future metabolic and physiological function of the offspring, given the established association between reduced fetal and placental growth and increased future risk of obesity and poor metabolic health [46–48]. However, further studies are required in order to determine the longer-term impacts of maternal dietary LA;ALA ratio and total fat load on the growth, body composition and cardiometabolic health of the offspring. Human studies that have assessed the potential implication of a higher omega-6:omega-3 ratio and/or elevated maternal omega-6 intakes on growth, body composition or metabolic health of the children are also limited [49] and further studies (or re-evaluation of existing studies) are needed. The results of the current study have also demonstrated that at least some of the effects of maternal dietary fat load/LA:ALA ratio elicited sex-specific responses, which may in part be due to differences in the placental response to omega-3 LCPUFA between male and female placentas. Further research is required to assess the placental responses to shifts in maternal dietary fat content and composition in both males and females and the “sex of the placenta” should be considered in future studies.

## Abbreviations

AA: Arachidonic acid
*Actb*: Beta-actin
ALA: Alpha-linolenic acid
BMI: Body mass index
BW: Body weight
DBS: Dried blood spot
DHA: Docosahexaenoic acid
EPA: Eicosapentaenoic acid
FAME: Fatty acid methyl esters
*Fasn*: Fatty acid synthase
FID: Flame ionization detector
GC: Gas chromatograph
LA: Linoleic acid
LCPUFA: Long chain polyunsaturated fatty acid
*Lep*: Leptin
*Lpl*: Lipoprotein lipase
MUFA: Monounsaturated fatty acid
*Pparg*: Peroxisome proliferator-activated receptor gamma
PUFA: Polyunsaturated fatty acid
RT-qPCR: Reverse transcription quantitative real-time polymerase chain reaction
SFA: Saturated fatty acid
*Srebf1*: Sterol regulatory element binding protein (variant 1c)
TFA: Trans fatty acid
VEGF: Vascular endothelial growth factor
